# Educational roles as a continuum of mentoring’s role in medicine – a systematic review and thematic analysis of educational studies from 2000 to 2018

**DOI:** 10.1186/s12909-019-1872-8

**Published:** 2019-11-27

**Authors:** Lalit Kumar Radha Krishna, Yaazhini Renganathan, Kuang Teck Tay, Benjamin Jia Xing Tan, Jia Yan Chong, Ann Hui Ching, Kishore Prakash, Nicholas Wei Sheng Quek, Rachel Huidi Peh, Annelissa Mien Chew Chin, David C. M. Taylor, Stephen Mason, Ravindran Kanesvaran, Ying Pin Toh

**Affiliations:** 1 Division of Supportive and Palliative Care, National Cancer Centre Singapore, 11 Hospital Drive, Singapore, 169610 Singapore; 2 Yong Loo Lin School of Medicine, National University of Singapore, Singapore, Singapore; 3 Palliative Care Institute Liverpool, Academic Palliative & End of Life Care Centre, University of Liverpool, North West Cancer Research Centre, Liverpool, UK; 4 Centre for Biomedical Ethics, National University of Singapore, Singapore, Singapore; 5 Duke-NUS Graduate Medical School, Singapore, Singapore; 6 Singapore General Hospital, Singapore, Singapore; 7 Medical Library, National University of Singapore Libraries, National University of Singapore, Singapore, Singapore; 8 Gulf Medical University, Ajman, United Arab Emirates; 9 Department of Medical Oncology, National Cancer Centre Singapore, Singapore, Singapore; 10 Department of Family Medicine, National University Health System, Singapore, Singapore

**Keywords:** Mentoring, Medicine, Supervision, Coaching, Role model, Undergraduate, Postgraduate

## Abstract

**Background:**

Recent studies have gone to great lengths to differentiate mentoring from **teaching, tutoring, role modelling, coaching and supervision** in efforts to better understand mentoring processes. This review seeks to evaluate the notion that **teaching, tutoring, role modelling, coaching and supervision** may in fact all be part of the mentoring process. To evaluate this theory, this review scrutinizes current literature on **teaching, tutoring, role modelling, coaching and supervision** to evaluate their commonalities with prevailing concepts of novice mentoring.

**Methods:**

A three staged approach is adopted to evaluate this premise. Stage one involves four systematic reviews on one-to-one learning interactions in **teaching, tutoring, role modelling, coaching and supervision** within Internal Medicine, published between 1st January 2000 and 31st December 2018. Braun and Clarke’s (2006) approach to thematic analysis was used to identify key elements within these approaches and facilitate comparisons between them.

Stage two provides an updated view of one-to-one mentoring between a senior physician and a medical student or junior doctor to contextualise the discussion.

Stage three infuses mentoring into the findings delineated in stage one.

**Results:**

Seventeen thousand four hundred ninety-nine citations were reviewed, 235 full-text articles were reviewed, and 104 articles were thematically analysed. Four themes were identified – characteristics, processes, nature of relationship, and problems faced in each of the four educational roles.

**Conclusions:**

**Role modelling, teaching and tutoring, coaching and supervision** lie within a mentoring spectrum of increasingly structured interactions, assisted by assessments, feedback and personalised support that culminate with a mentoring approach. Still requiring validation, these findings necessitate a reconceptualization of mentoring and changes to mentor training programs and how mentoring is assessed and supported.

## Background

Mentoring nurtures professional and personal development [1], improves learning and clinical competency and enhances career satisfaction amongst mentees and mentors [2, 3]. These successes are largely reliant upon the mentor’s ability to nurture personalized mentoring relationships and steer the mentoring process [2–16]. To do so, mentors adopt many supportive and educational roles [2–16]. These include being a supervisor “*focused upon professional development of the student”,* a coach *facilitating learner development through use of “deliberate practice strategies”,* a role model “*setting out to create a positive example of good practice”,* an advisor “*helping with scheduling, logistics and applications”* and a sponsor “*influencing promotion and advancement*” [2–16]. This has fuelled the notion that **teaching, tutoring, role modelling, coaching and supervision** lie within the scope of a mentoring role and dismissed long-held beliefs that conflation between these practices and mentoring was a significant source of confusion in conceptualizing mentoring. To better understand this perspective, a clear understanding of **teaching, tutoring, role modelling, coaching and supervision** is warranted. This requires distancing these practices from prevailing accounts of mentoring that is often conflated with these supportive and educational roles [2–16]. Here mentoring is defined as a ‘*dynamic, context dependent, goal sensitive, mutually beneficial relationship between an experienced clinician and junior clinicians and or undergraduates that is focused upon advancing the development of the mentee’* [2–16].

### The need for this study

The implications of this theory would necessitate a review of how mentoring is conceived and have wide-ranging effects upon the understanding, structuring, oversight and support of mentoring approaches, curricula and mentor training programs [2–16].

## Methods

To evaluate the notion that **teaching, tutoring, role modelling, coaching and supervision** may be a part of an overarching concept of mentoring, this study was made up of three stages. Stage 1 consists of systematic reviews of **teaching, tutoring, role modelling, coaching and supervision** carried out to provide better understanding of these processes. In acknowledgement of mentoring’s, coaching’s and supervision’s context-dependent, approach-specific nature, studies were confined to educational accounts that involve one-to-one interactions between tutor and learner. The term tutor was used to encapsulate mentor, supervisor, teacher, role model and coach.

Stage 2 drew upon prevailing descriptions of novice mentoring, the dominant form of mentoring, given that mentoring’s context dependent nature prevents conflation of different forms of mentoring [2–16].

Stage 3 sought to determine similarities between **teaching, tutoring, role modelling, coaching and supervision** and mentoring.

To carry out the systematic reviews, Stage 1 adopted Braun and Clarke’s (2006) approach to thematic analysis to identify key themes within **teaching, tutoring, role modelling, coaching and supervision** in medical education [17].

Stage 2 drew upon recent accounts of novice mentoring. Focus was maintained on novice mentoring which is the dominant form of mentoring in medical education and to prevent it from being conflated with other distinct forms of mentoring such as peer, group and e-mentoring [2–16].

Stage 3 sought comparisons being made between novice mentoring and **teaching, tutoring, role modelling, coaching and supervision** to determine the overlap between each of these approaches.

### Stage 1: thematic analysis of teaching, tutoring, role modelling, coaching and supervision

#### Methodology

A systematic review was proposed to explore the size and scope of available literature on assessing the impact of medical ethics education in published peer-reviewed literature [18–22]. This allowed for systematic extraction and synthesis of actionable and applicable information [23] whilst summarizing available literature [24, 25] across a wide-range of pedagogies, assessment contents and practice settings [26–30].

Levac et al. (2010) [31]‘s and Arksey and O’Malley (2005) [18]‘s framework for systematic review was used to map “the key concepts underpinning a research area and the main sources and types of evidence available” [21] and “produce a profile of the existing literature in a topic area, creating a rich database of literature that can serve as a foundation” to inform practice and guide further research [19, 32, 33].

Guided by PRISMA-P 2015 checklist [24], Levac et al. (2010) [31]‘s and Arksey and O’Malley (2005) [18]‘s framework, a 6-stage systematic review protocol was developed for this study [18–22, 31].

##### Stage a: identifying the research question

Guided by two librarians from the medical libraries at Yong Loo Lin School of Medicine at National University Singapore and the National Cancer Centre Singapore and educational experts and clinicians from the Singapore General Hospital, the Division of Cancer Education at the National Cancer Centre Singapore and the Marie Curie Palliative Care Institute at the University of Liverpool (henceforth the advisory team), the 14-person research team (YR, JY, AH, KT, KP, NQ, RP, BT, AC, YP, RK, DT, SM, and LK) discussed prevailing concepts and practice surrounding issues, and practices surrounding **teaching, tutoring, role modelling, coaching and supervision** and identified the primary research question to be: ‘what is known of **teaching, tutoring, role modelling, coaching and supervision** in Internal Medicine?’. The secondary questions were “what are the key elements of **teaching, tutoring, role modelling, coaching and supervision** in Internal Medicine?’. These questions were designed on the population, concept and context elements of the inclusion and exclusion criteria [34, 35], using a PICOS format (Table 1).

**Table 1:** PICOS, inclusion and exclusion criteria applied to literature search

	Role modelling		Teaching and tutoring		Coaching		Supervision	
PICOS	Inclusion criteria	Exclusion criteria	Inclusion criteria	Exclusion criteria	Inclusion criteria	Exclusion criteria	Inclusion criteria	Exclusion criteria
Population	- Postgraduate or undergraduate - Involve medical student/ junior clinician/ resident and/or senior clinician in a facility where medical services are provided, or in a facility of medical education - Within clinical or academia or research setting - Limited to Internal Medicine and General Medicine	- Clinical specialties not associated with Internal Medicine such as Surgery, Paediatrics, Emergency Medicine, Psychiatry, Obstetrics and Gynaecology, and Clinical and Translational Science - Allied health roles, such as Nursing, Physiotherapy, Occupational Therapy, Pharmacy, Radiography, Psychology, Medical Social Work - Wet bench research/lab work - Veterinary work or Dentistry - Ancillary staff such as housekeepers, administrators, finance professionals, accountancy	- Postgraduate or undergraduate - Involve medical student/ junior clinician/ resident and/or senior clinician in a facility where medical services are provided, or in a facility of medical education - Within clinical or academia or research setting - Limited to Internal Medicine and General Medicine	- Clinical specialties not associated with Internal Medicine such as Surgery, Paediatrics, Emergency Medicine, Psychiatry, Obstetrics and Gynaecology, and Clinical and Translational Science - Allied health roles, such as Nursing, Physiotherapy, Occupational Therapy, Pharmacy, Radiography, Psychology, Medical Social Work - Wet bench research/lab work - Veterinary work or Dentistry - Ancillary staff such as housekeepers, administrators, finance professionals, accountancy	- Postgraduate or undergraduate - Involve medical student/ junior clinician/ resident and/or senior clinician in a facility where medical services are provided, or in a facility of medical education - Within clinical or academia or research setting - Limited to Internal Medicine and General Medicine	- Clinical specialties not associated with Internal Medicine such as Surgery, Paediatrics, Emergency Medicine, Psychiatry, Obstetrics and Gynaecology, and Clinical and Translational Science - Allied health roles, such as Nursing, Physiotherapy, Occupational Therapy, Pharmacy, Radiography, Psychology, Medical Social Work - Wet bench research/lab work - Veterinary work or Dentistry - Ancillary staff such as housekeepers, administrators, finance professionals, accountancy	- Postgraduate or undergraduate - Involve medical student/ junior clinician/ resident and/or senior clinician in a facility where medical services are provided, or in a facility of medical education - Within clinical or academia or research setting - Limited to Internal Medicine and General Medicine	- Clinical specialties not associated with Internal Medicine such as Surgery, Paediatrics, Emergency Medicine, Psychiatry, Obstetrics and Gynaecology, and Clinical and Translational Science - Allied health roles, such as Nursing, Physiotherapy, Occupational Therapy, Pharmacy, Radiography, Psychology, Medical Social Work - Wet bench research/lab work - Veterinary work or Dentistry - Ancillary staff such as housekeepers, administrators, finance professionals, accountancy
Intervention	- Accounts of, or involving, role modelling between medical student, junior clinician, and/ or resident, and senior clinician - Dyadic/ One-to-one role modelling relationship - Role modelling process - Characteristics of the role model and student - Role modelling relationship - Barriers of role modelling	- Supervision, coaching, teaching, tutoring, advising and sponsorship - Peer mentoring, mentoring for leadership, mentoring patients or mentoring by patients, interdisciplinary mentoring - No mention of role modelling	- Accounts of, or involving, senior and junior clinicians, residents and/or medical students who underwent tutoring and/or teaching - Dyadic/ One-to-one teaching/ tutoring relationship - Teaching/ tutoring process - Characteristics of the teacher/ tutor and student - Teaching/ tutoring relationship - Barriers of teaching/ tutoring	- Supervision, coaching, role modelling, advising and sponsorship - Peer mentoring, mentoring for leadership, mentoring patients or mentoring by patients, interdisciplinary mentoring - No mention of teaching or tutoring	- Accounts of, or involving, coaching between medical student, junior clinician, and/ or resident, and senior clinician - Dyadic/ One-to-one coaching relationship - Coaching process - Characteristics of coach and student - Coaching relationship - Barriers of coaching	- Supervision, role modelling, teaching, tutoring, advising and sponsorship - Peer mentoring, mentoring for leadership, mentoring patients or mentoring by patients, interdisciplinary mentoring - No mention of coaching	- Accounts of, or involving, supervision between medical student, junior clinician, and/ or resident, and senior clinician - Dyadic/ One-to-one supervising relationship - Supervising process - Characteristics of supervisor and student - Supervising relationship - Barriers of supervision	- Supervision of procedure - Role modelling, coaching, teaching, tutoring, advising and sponsorship - Peer mentoring, mentoring for leadership, mentoring patients or mentoring by patients, interdisciplinary mentoring - No mention of supervision
Comparison	Comparisons of accounts on dyadic role modelling, including its approach to implement dyadic role modelling, processes, characteristics, challenges, evaluation methods and criteria		Comparisons of accounts on dyadic tutoring and teaching, including its approach to implement dyadic teaching/tutoring, processes, characteristics, challenges, evaluation methods and criteria		Comparisons of accounts on dyadic coaching, including its approach to implement dyadic coaching, processes, characteristics, challenges, evaluation methods and criteria		Comparisons of accounts on dyadic supervision, including its approach to implement dyadic supervision, processes, characteristics, challenges, evaluation methods and criteria	
Outcome	- Personal outcomes - Professional outcomes - Career-related outcomes - Research and academia outcomes - Impact on role model and student	Studies where role modelling outcome was not the main component studied	- Personal outcomes - Professional outcomes - Career-related outcomes - Research and academia outcomes - Impact on teacher/ tutor and student	Studies where teaching/ tutoring outcome was not the main component studied	- Personal outcomes - Professional outcomes - Career-related outcomes - Research and academia outcomes - Impact on coach and student	Studies where coaching outcome was not the main component studied	- Personal outcomes - Professional outcomes - Career-related outcomes - Research and academia outcomes - Impact on supervisor and student	Studies where supervising outcome was not the main component studied
Study design	All study designs are included: - Descriptive papers - Qualitative, quantitative and mixed study methods - Published between 1 Jan 2000 and 31 Dec 2018 - Written in English Language or translated into English Language	- Grey literature - Perspectives, opinion, commentary pieces and editorials	All study designs are included: - Descriptive papers - Qualitative, quantitative and mixed study methods - Published between 1 Jan 2000 and 31 Dec 2018 - Written in English Language or translated into English Language	- Grey literature - Perspectives, opinion, commentary pieces and editorials	All study designs are included: - Descriptive papers - Qualitative, quantitative and mixed study methods - Published between 1 Jan 2000 and 31 Dec 2018 - Written in English Language or translated into English Language	- Grey literature - Perspectives, opinion, commentary pieces and editorials	All study designs are included: - Descriptive papers - Qualitative, quantitative and mixed study methods - Published between 1 Jan 2000 and 31 Dec 2018 - Written in English Language or translated into English Language	- Grey literature - Perspectives, opinion, commentary pieces and editorials

**Fig. 1:**
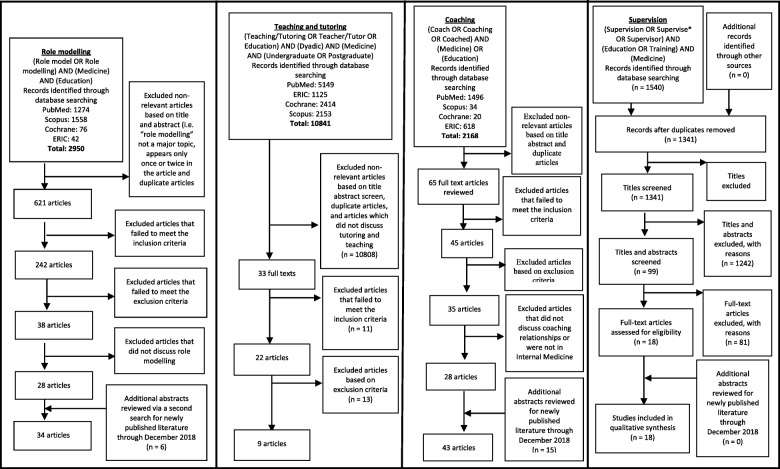
PRISMA search results and selection

Guided by the advisory team, the 14-person research team worked in teams of threes under the supervision of the senior researchers (LK, SM, DT, and RK) and supported by near peer mentors (YP and KT) to carry out independent searches of accounts of role modelling, teaching, tutoring, coaching and supervision published in the PubMed, Scopus, ERIC and Cochrane Database of Systematic Reviews. The searches were carried out between the 12th September 2017 and 18th October 2017. The respective search strategies are found in the PRISMA in Fig. 1. In keeping with Pham et al. (2014) [33]‘s approach of ensuring a viable and sustainable research process, articles published in English or had English translations published between 1st January 2000 to 31st December 2015 were included in the initial search.

With all searches reviewed by the senior reviewers, the review process was extended, and additional searches were performed between 12th May 2019 and 24th April 2019 to review newly published literature from 1st January 2016 to 31st December 2018 for each of the learning approaches.

Focus was on accounts of role modelling, tutoring, teaching, coaching and supervision that clearly described one-on-one interactions between a clinician and a learner in Internal Medicine. Accounts of **teaching, tutoring and role modelling** that did not clearly state one-on-one interactions were excluded as it did not facilitate comparisons with mentoring, supervision and coaching. Accounts of **teaching, tutoring, role modelling, coaching and supervision** in clinical specialities not traditionally associated with Internal Medicine as defined by the World Health Organization’s classification of healthcare workers, were also excluded to further focus this review [36].

Braun and Clarke’s (2006) approach to thematic analysis [17] was used to circumnavigate the wide-range of research methodologies that made statistical pooling and analysis difficult [17] in the papers reviewed. The narrative produced was guided by the Best Evidence Medical Education (BEME) Collaboration guide [37] and the STORIES (Structured approach to the Reporting In healthcare education of Evidence Synthesis) statement [38].

##### Stage B: identifying relevant studies

Guided by the advisory team, the research team developed individual search strategies for **teaching, tutoring, role modelling, coaching and supervision** and selected PubMed, Embase, PsycINFO, and ERIC databases for review. In keeping with Pham et al. (2014) [33]‘s approach of ensuring a viable and sustainable research process, the research team confined the searches to articles published between 1 January 2000 and 31 December 2018 to account for prevailing manpower and time constraints faced by the team.

##### Stage C: selecting studies to be included in the review

After the independent searches of the databases were combined employing the ‘negotiated consensual validation’ approach and a final list of article to be reviewed was determined, the 7-members of the research team (YR, JY, AH, KP, NQ, RP, BT) guided by the senior reviewers (SM, RK, DT and LK) and near peer mentors (KT and YP) independently screened the title and abstracts.

A consensus based approach employing the ‘negotiated consensual validation’ approach was reached on the final list of papers to be included for thematic analysis [39].

The PRISMA charts are attached below (Fig. 1).

##### Stage D: data characterization and analysis

In the absence of a priori framework and a clear definition of role modelling, teaching and tutoring, coaching and supervision, Braun and Clarke’s (2006) [17] approach to thematic analysis was adopted to identify consistencies across these approaches [2, 14, 15, 17, 40–44].

Braun and Clarke’s (2006) approach was used to create codes from the ‘surface’ meaning of the data. Semantic themes were identified from ‘detail rich’ codes focused upon the various aspects of the role modelling, teaching, tutoring, coaching and supervisory process [17]. Each of the 10 coded scripts from role modelling, teaching and tutoring, coaching and supervision were reviewed by the senior reviewers. The research team discussed and agreed upon a common coding framework and codebook using Sambunjak et al. (2010)‘s “negotiated consensual validation” approach [45]. Working in teams of three, overseen by the senior reviewers (SM, RK, DT, and LK) and peer mentors (KT and YP), the reviewers carried out independent thematic analyses of all articles in each of the four topics using the codebook, with new codes discussed online and at face-to-face at reviewers’ meetings [17, 46–49].

##### Stage E: collating, summarizing, and reporting the results

From the 18,938 articles reviewed, 34 articles on role modelling, 9 articles on teaching and tutoring, 43 articles on coaching and 18 articles on supervision were identified. The four themes identified include characteristics, processes, nature of relationship, and problems of the four educational roles.

The narrative produced was guided by the Best Evidence Medical Education (BEME) Collaboration guide [37] and the STORIES (Structured approach to the Reporting In healthcare education of Evidence Synthesis) statement [50].

## Results

### Characteristics of each of the four educational roles

Thematic analysis of the prevailing descriptions of role modelling, teaching, tutoring, coaching and supervision were carried out. Their characteristics and descriptions are highlighted in the table below (Table 2).

**Table 2:** Characteristics of the four educational roles

Characteristics and descriptions	References
Role Modelling	
Webster’s Dictionary “a person considered as a standard of excellence to be imitated.”	[51–53]
Combination of personal characteristics (Heart), professional patient care (Hands-on), and teaching that involves continuously making the implicit explicit (Head). Being a role model, as opposed to being a teacher or a mentor when the moment calls for it, implies that the clinical trainer integrates the “3Hs” as a unity all the time and everywhere.	[54]
everything faculty do in their being and acting as professionals both inside and outside the hospital	[55–58]
Specific observable behaviour (as well as attitudes and values) to be emulated or even surpassed by residents	[58–62]
Role modelling has been defined as “a way responses (specific observable behaviour as well as attitudes and values) can be learned or weakened through exposure to significant others”.	[62]
Demonstration of clinical skills, modelling and articulation of expert thought processes and manifestation of positive professional characteristics	[63]
Interactional, transactional process, which occurs simultaneously with multiple models and changes over time.	[64]
Teaching and Tutoring	
Clinical skills training and knowledge transfer	[65–71]
Professional outcome-based assessment	[66, 70, 71]
Feedback provided for students after teaching or tutoring	[65, 66]
Standardized program structure	[67, 68]
Coaching	
Individualized	[72–81]
Safe space for coached to make mistakes and learn	[73, 76, 77, 82, 83]
Deliberate teaching with focused goals	[75, 82, 84–86]
Individualized feedback through observation	[74–77, 79, 81–91]
Repetition	[82, 87, 89, 90]
Supervision	
Apprenticeship	[92]
Clinical care under the oversight of a more senior physician	[92]
Improve resident education through identifying trainee problems, provision of feedback and supporting trainee	[93–98]
Ensure effective and safe patient care	[92, 93, 99]

### Educational processes

Role modelling is often a ‘one off’ unstructured experience that neither the learner nor the role model has prepared for [100]. Role modelling may be unconsciously carried out and may be either an exemplary action or one that ought not to be repeated [55, 57, 59, 62]. Given the unplanned nature of role modelling, it may even breach standards of practice [55, 57, 59, 62]. Role modelling may not have a longitudinal component and is often not appraised [55, 57, 59, 62], subject to feedback or reflection [60, 63, 100–102].

The learning processes in teaching and tutoring, coaching and supervision are interactive [64–66, 74–77, 79, 81–91, 93–98], context-specific, goal-sensitive and dynamic [63, 75, 82, 84–86, 92] process that are guided by the objectives of the clinical training program [66, 70, 71, 92, 93, 99] and supported and overseen by a host organization [67, 68, 73, 76, 77, 82, 83, 92].

Teaching and tutoring need not be matched though having learners and tutors with complementary abilities, motivations, personalities, and values for coaching and supervision is helpful. Teaching and tutoring, coaching and supervision is influenced by individual learning goals [75, 82, 84–86], relationships [72–81], program structure [67, 68], assessments [66, 70, 71], and environment [64]. All teaching and tutoring, coaching and supervision programs are structured, planned, and often have longitudinal component which are horizontally and vertically integrated [90, 103, 104]. The programs usually include feedback, reflection and an evaluative process [90, 103, 104].

**Fig. 2:**
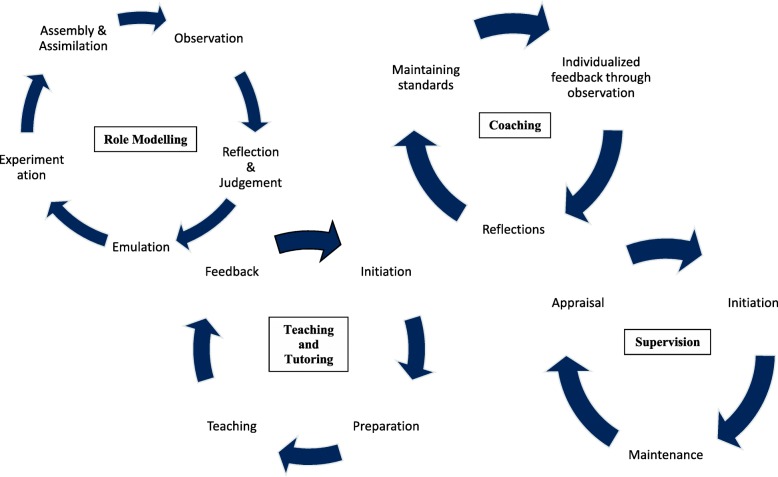
Learning processes across different educational approaches

The distinctive aspects of each learning processes are highlighted in Fig. 2.

### Nature of relationship

#### Role Modelling 

Role modelling is unpredictable and involves varying levels of interaction and communication [100]. Some interactions are purposive; built through sharing of professional and personal experiences and socializing [60, 63, 101, 102] whilst others are entirely opportunistic.

A lack of structure however may result in negative role modelling [55, 57, 59, 62]. Negative role modelling [55, 57, 59, 62] may dissuade students from particular career choices [57], cause trainees to become cynical about academic life [62], discourage reflective practice skills [55] and undermine professional and patient-centered behaviour [63].

#### Teaching and tutoring

Structured programs [66] that include experiential learning [65–71], discussions [70, 71], or guided reflections [65, 66] formed the basis for teacher-student interactions [65, 70]. Given the variability of these interactions [68, 69, 71, 105], teacher-student relationships tend to be superficial [65, 66]. However, supportive and approachable teachers [65–67, 106], who are willing to commit and provide student-centric teachings [65, 66, 70, 105], are able to develop more successful learning relationships and achieve greater goals [69, 70, 106].

#### Coaching

The relationship between the coach and trainee is focused upon learning a specific skill [72]. The complexity of the skill determines the duration of the relationship [72].

Coaching begins with the demonstration of the skills in a planned role modelling process, which tapers over time as goals were achieved and as trainees develop their ability to self-monitor and sustain their training [72].

It is debatable as to whether coaching provides psychological and emotional support [74, 80, 84]. Some commentators suggest that coaching relationships are transactional and focused upon professional improvement whilst others suggest the presence of evaluations within coaching interactions necessitate a trusting [74, 80, 84] and safe environment [73, 90, 91].

#### Supervision

The supervisory relationships are hierarchical [92]. With the trainee dependent on the supervisor for academic progression and career advancement [92], trusting relationships between supervisor and trainee are less likely [92–99].

A comparison of the nature of relationships across various educational roles are found in Appendix 1 in Table 4.

### Problems faced in the four educational roles

Each of these educational approaches face common problems. Many revolve around insufficient training, poor program structure, inadequate learning resources and inaccurate program evaluation and learning assessment [55, 80, 93, 95, 96, 100, 107–111].

Role modelling faces limited time for teaching [55, 100, 107–109] and bedside tutorials [110] whilst coaching faces inadequate financial, administrative and assessment support that are not conducive of nurturing organizational culture to ensure protected time and recognition for coaches [80, 111].

Supervision faces organizational issues that include a lack of consistent level of support and training [93], resource limitation and competing tensions between service and education demands [95, 96]. A detailed account of these challenges is found in Appendix 2 in Table 5.

## Discussion

### Drawing the findings together

Based on the data from the four systematic reviews, it is possible to proffer a clearer understanding of each of the approaches.

#### Role Modelling

Positive role modelling can be defined as “*a process where a trainer consciously or unconsciously demonstrates positive or negative behaviours, actions or attitudes. The learner observes, weighs up and reflects upon these characteristics, skills and or behaviours upon their own practice/attitude/behaviour and emulates, experiments, and assimilates it into his/her own personal/professional identity. Positive role modelling is more impactful when it occurs in a trusting, professional relationship.”* [51–55, 57–64, 100, 102, 109, 112–115]

#### Teaching and tutoring

Teaching and tutoring is *“a professional goal-specific* [66]*, task-oriented* [66]*, standardized* [67, 68]*, and structured learning process* [66, 69] *on clinical knowledge and skills* [65–71]*, driven by clinical competency and performance outcomes* [69, 70, 106]*. The professional* [65, 66, 69–71, 105, 106]*, tutor-, and student-dependent* [65, 66] *tutor-learner relationship requires protected time* [65–67, 70] *to develop in a safe and productive learning environment* [69]*, supported by the host organization* [67]*, for effective teaching* [65, 67, 69–71, 106] *and feedback* [66, 67, 70, 71, 105] *processes.”*

#### Coaching

Coaching can be defined as a “*longitudinal professional relationship between an expert coach and a trainee focused upon mastery of a clearly defined, measurable and achievable skill that is that the trainee or training organization feels the trainee can improve upon. The relationship is built upon professional trust in a ‘safe environment’ that facilitates practice of the skill. The coach evaluates the performance, needs and abilities of the trainee, role models skills, encourages learning, provides specific individualized feedback and devises a plan to achieve the goals. The trainee is accountable for their training and responsible for self-monitoring.*” [73–86, 88–91]

#### Supervision

Supervision is an “*individualized, focused, goal-specific, time-limited and context-sensitive clinical training process by a senior clinician aimed at assessing and improving particular gaps and weaknesses in the clinical care and patient safety by trainees by providing them with oversight, guidance and feedback and holding trainees up and accountable to established clinical standards and codes of practice.* This process will utilize coaching and role modelling to meet its goals [92–99].

**Fig. 3:**
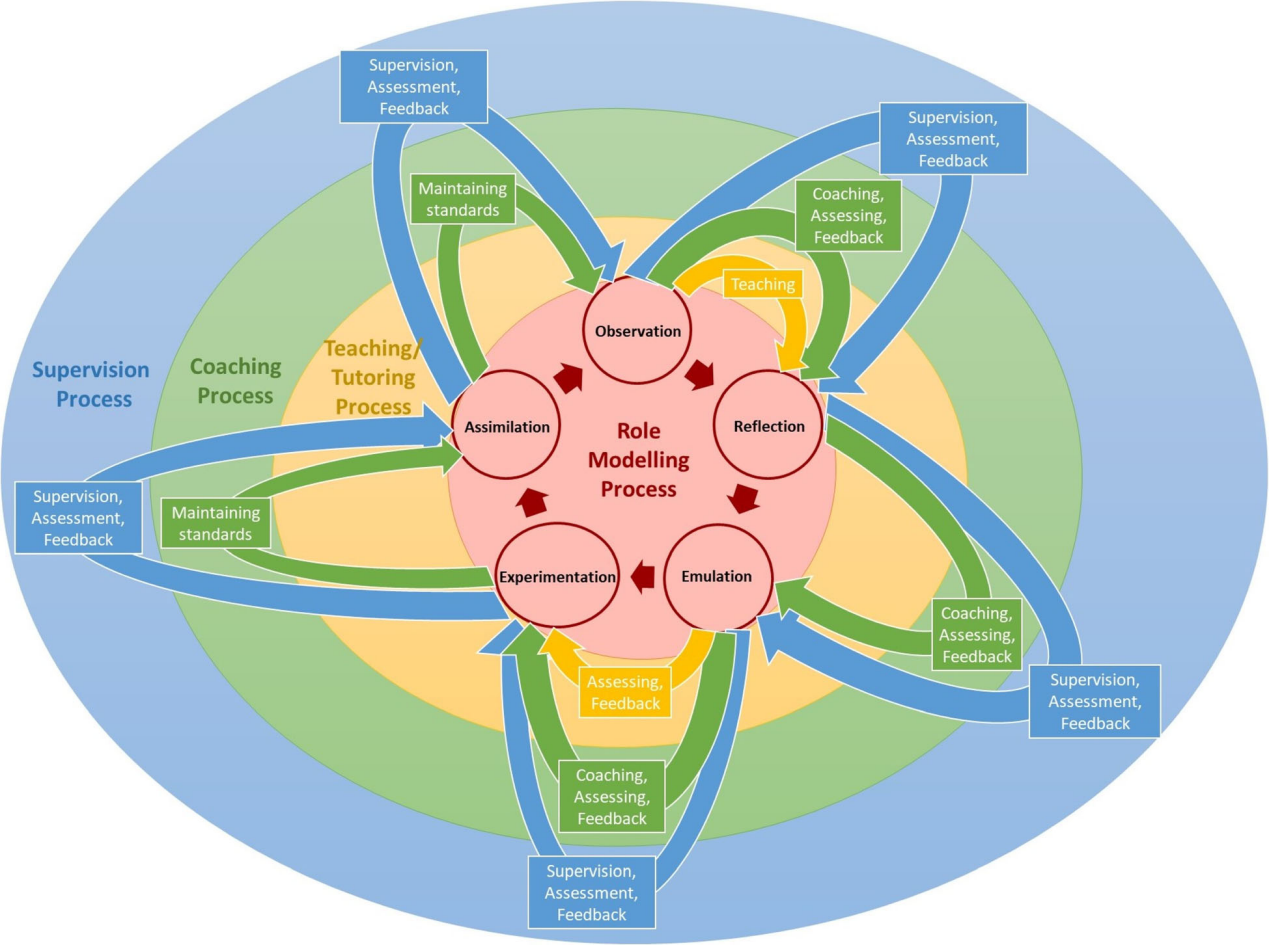
The relationship between the four approaches

Comparing the findings of the four systematic reviews, there are a number of key insights and similarities that may be discerned. These commonalities lay the foundation for a collective perspective of the four educational approaches. These features are shown in Table 3 and the characteristics of each approach is shown in Fig. 3.

**Table 3:** Features of the role modelling, teaching and tutoring, coaching and supervision

Features	Role model	Teaching and tutoring	Coach	Supervision
Planned	No	Yes	Yes	Yes
Matching	No	Equivocal	Yes	Yes
Structure	No	Yes	Yes	Yes
Positive/Negative Exemplar	Both	Positive	Positive	Positive
Assessment	No	Yes	Yes	Yes
Feedback	No	Maybe	Yes	Yes
Context sensitive	No	Yes	Yes	Yes
Goal specific	No	Yes	Yes	Yes
Bilateral/dynamic interaction	No	Yes	Yes	Yes
Longitudinal	No	Yes	Yes	Yes
Integrated	No	Yes	Yes	Yes
Reflection	No	Yes	Yes	Yes
Type of relation	Superficial	Superficial	Trusting	Trusting/deep
Tutor dependent	Maybe	Yes	Yes	Yes
Specific	No	Yes	Yes	Yes
Practice	No	Yes	Yes	Yes
Psycho-emotional support	No	Yes	Yes	Yes

The data would suggest that the more relevant aspects of role modelling appears to be contained within teaching and tutoring, which in turn appears to be subsumed by coaching. Supervision appears to contain features of role modelling, teaching and tutoring, and coaching.

Viewed figuratively as concentric rings, role modelling would be at the centre of the rings, enclosed by teaching and tutoring, then coaching and finally supervision as the outer most ring.

### Stage 2 mapping mentoring practice

To determine mentoring’s relationship with the concentric rings featured in Fig. 3, Stage 2 will provide a summary of prevailing concepts of mentoring drawn from two recent systematic scoping reviews of novice mentoring and a recent study of mentoring experiences within a novice mentoring program.

Sng et al. (2017) [2]‘s and Tan et al. (2018) [12]‘s systematic scoping reviews highlight a number of key aspects of mentoring

1. Mentoring can be defined as ‘*dynamic, context dependent, goal sensitive, mutually beneficial relationship between an experienced clinician and junior clinicians or undergraduates that is focused upon advancing the development of the mentee*’.
2. Mentoring possesses adopt an evolving, adaptive, goal-specific, context-sensitive, and mentee-, mentor-, relationship-, and host organization-dependent nature (mentoring’s nature) that prevents conflation with other forms of mentoring.
3. Novice mentoring’s success lies with its nurturing of personalized relationships between the mentee and mentor
4. To develop personalized mentoring relationships, there must be balance between individualization of mentoring relationships that includes catering to the mentee’s needs, abilities, goals and situation and ensuring a consistent mentoring approach that is both compliant to prevailing codes of conduct and sufficiently structured to allow effective, timely, appropriate, personalized, specific, holistic, longitudinal and accessible evaluations and support for the mentee, mentoring and the mentoring relationship.

Evidencing these findings and forwarding new insights of novice mentoring, Krishna et al. (2019) [3]‘s study of mentoring experiences in a novice mentoring program also unearthed new aspects to mentoring. These include

1. mentoring’s competency-based stages of development that requires mentees to achieve basic competencies at each stage of the mentoring process before progressing to the next stage.
2. progress through the various stages of mentoring requires effective communication, timely and appropriate assessments and support appropriate balancing between consistency and structure.
3. oversight and support of the mentoring process depends upon the host organization and well-trained and supported mentors.

### Stage 3 mentoring Spectrum

**Fig. 4:**
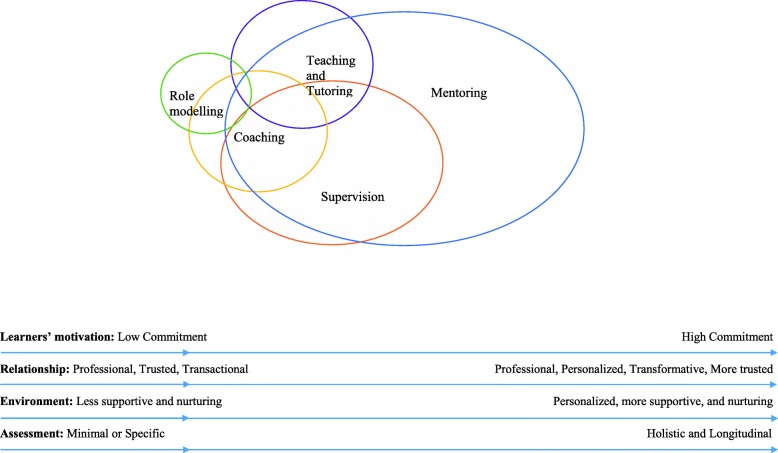
Conceptual Framework of the Mentoring Continuum Model and Overarching concept of Mentoring

Mentoring’s use of personalized holistic and longitudinal support throughout the mentoring process would require mentees to be taught, and provided with guidance as they apply their knowledge and skills, be assessed and provided with feedback and then re-evaluated before progressing to the next stage of the mentoring process. At each stage of the mentoring process which Krishna et al. (2019) [3] describe as “*‘circumscribed sequential projects’ with ‘specific goals and competency requirements*’” , it is likely that mentors will employ role modelling, teaching and tutoring, coaching and supervision to support the mentee and the evolving mentoring relationship. This would see mentoring encompassing supervision’s role and occupying the outer most ring in Fig. 4.

This suggests that role modelling, teaching, tutoring, coaching and supervision lie within a **mentoring spectrum (**Fig. 4**).** The mentoring spectrum describes a range of educational practices contained under the aegis of mentoring beginning with role modelling on the left side of the spectrum and mentoring on the right. Beginning with role modelling, there is progressively complex interactions culminating with personalized attention in role modelling behaviours, attitudes and practices, teaching new skills and knowledge, coaching individual learner’s on different aspects of the skills they need, appraising their progress and providing feedback as they are supervised to complete their immediate goals within the project. Separating supervision from the more complex relationships seen in mentoring is the provision of personalized, timely, holistic and longitudinal support and the adaptation of the mentoring approach to accommodate the mentee’s needs, goals, circumstance and abilities.

Other features that evidence the notion of a mentoring spectrum include

1. *Motivation of learners*

All educational approaches are reliant upon the learner’s ability to observe, discern gaps in their ability, learn, reflect, weigh up considerations, be open to feedback and be accountable for their own learning. However, the approaches rely on increasing learner motivation moving from left to right of the mentoring spectrum.

2. *Learning Relationship*

Moving from left to right along the mentoring spectrum also highlights increasing planning and structuring of the mentoring process. Improved structuring of educational interactions better supports learning relationships and nurtures more holistic and personalised educational relationships. Better learning relationships also facilitate better outcomes.

Learning relationships also become more interactive moving from left to right in the mentoring spectrum. In role modelling, learners may not have an educational relationship with the tutor whilst learning relationships in mentoring are dynamic and enduring [2, 92, 116–119].

3. *Nurturing learning environment*

Building a learning relationship relies on the learning environment and moving from left to right of the mentoring spectrum sees learning environments becoming increasingly important to the quality and nature of the learning relationship. These learning environments also become more individualised and serve to nurture particular learning relationships within the larger educational environment. This is especially evident in supervision and mentoring [90, 91].

4. *Learning assessment*

Assessments also play an increasing role moving from left to right of the mentoring spectrum. These assessments must be timely, appropriate and personalised and accompanied by open and frank discussions and personalized, appropriate, specific, timely, holistic, accessible and longitudinal feedback and support [2, 116–119]. The presence of regular appraisals also reiterate the importance of individualized and safe educational environment [90, 91].

### The impact of the mentoring spectrum

An overarching mentoring spectrum combining role modelling, teaching and tutoring, coaching, supervision, and mentoring has wide ramification upon how these educational approaches are employed.

1. The implication upon mentor training is significant. Acknowledging the roles to be played within the mentoring spectrum highlights the need for mentors to be trained in all these educational approaches.
2. The unplanned and unconscious nature of role modelling and the need for balance between personalising and consistency within the mentoring approach both highlight the need for clear standards of practice, codes of conduct and practice guidelines (henceforth Codes of Practice or CoP). There must also be opportunities for mentee and mentors to align expectations and accept their responsibilities and roles and for mentees and mentors to be briefed on the prevailing goals and timelines of their respective educational projects and processes.
3. The learners and tutors must also be appropriately matched to ensure that they have complementary working styles, learning approaches and personalities, goals and abilities [120, 121]. This will help build better educational interactions.

The implications of the mentoring spectrum upon mentoring practice is vast and includes requiring

i. mentors-in-training to be trained and skilled on all these educational approaches and be mentored when applying these skill sets and competencies for each of the educational roles.
ii. mentors and mentees to be briefed on CoPs, expectations on roles, responsibilities and expectations and effective oversight, assessment, and support provided by the host organization.
iii. robust, longitudinal and holistic assessment processes in light of the changing nature of the mentoring process and the mentor’s roles and the presence of evolving mentoring relationships and different stages of the various aspects within the mentoring spectrum.
iv. the host organization to take an active role in overseeing and providing personalized, appropriate, specific, timely, holistic, accessible and longitudinal financial and administrative support in running and overseeing the mentoring process given the diverse processes within the mentoring spectrum [2, 116–119].
v. that the mentoring process is sufficiently structured to accommodate for the inevitable changes in the mentoring process without breaching the CoP.
vi. the need for a safe and nurturing working environment that will nurture trusting and enduring mentoring relationships that will not only enhance better role modelling when the mentee has established ties with the mentor but also facilitate discussions that extend beyond professional issues which will allow the provision of holistic support.
vii. the need for an open and safe mentoring culture that allows open discussions, constructive feedback and frank discussions.

### Limitation

This review posits that these practices are interrelated is based on a number of novel yet unproven assumptions. Selecting only four of the many educational roles also limits the scope of understanding of the entire spectrum of educational roles in mentoring. In addition, the practices described in this review focus specific education settings, and draw from a particular definition of role modelling, teaching and tutoring supervision and coaching that may not be applicable in other education settings. Within the context of role modelling for example, there is no consideration of negative role modelling which limits the validity of the conclusions reached. In addition, many of the papers contextualized within the European and American healthcare system and training programs, limit their applicability to other educational and healthcare systems.

## Conclusions

The findings of this review not only suggest a new way of conceptualizing mentoring but also highlights the need for further study into the matching, pre-mentoring, mentoring relationship, mentoring evaluations, mentoring structure, the mentoring environment and mentoring culture. This theoretical concept though supported by data from novice mentoring processes will still need to be carefully studied and validated. One key area for further study must be the manner that mentees, mentors and the host organization interact (mentoring dynamics) given its influence upon all processes within the mentoring spectrum. Similarly, important is the design of effective assessment tools and policing of the mentoring process and the mentoring environment.

However, we are confident that this new concept of mentoring will enhance the mentoring process and mentoring outcomes as medical education strives toward personalized medical education.

## Data Availability

Raw data generated or analysed in this study can be provided upon request.

## Appendix 1

**Table 4:** Nature of learning relationship across various educational roles

	Role modelling	Teaching and tutoring	Supervision – breadth	Coaching – depth	Mentoring
Level of **commitment** involved	Relatively minimal: usually unaware and passive in practice, but effective when intentional and active	Intermediate – time needed for teaching relationship to form	Intermediate – ensure patient safety + resident development	Intermediate – ensure mastery of skills	High – psychosocial support as well as professional support
Type of commitment	Voluntary/involuntary	Voluntary/involuntary	Voluntary/involuntary	Voluntary/involuntary	Voluntary
Nature of **trust**	Professional				Professional and personal
Task and interpersonal balance	Either or both	Task-oriented			Balanced
Key to successful interaction/ relationship	Display of positive attributes	Safe and productive learning environment: Trusting and proactive, protected teaching time trusted by teacher	Safe space for learning, balance of trainees educational development and patient safety	Safe space for practicing skill to attain mastery, non-evaluative role	Personal connection built on shared values, mutual respect, commitment and trust
Duration of interaction	Episodic and random	Variable length depending on curriculum planning, from one session to a few years	Until supervisor is confident of supervisees’ skills Time limited, current	Until coach attains mastery of goal Time limited, current	Lifelong – evolves into friendship, gain colleague/Peer Long-term, future oriented
Transactional nature of relationship	-	Performance and professional learning outcomes driven			
Psychosocial Support	-	Providing constructive feedback about professional competency			Providing constructive feedback about professional competency As well as personal issues
Control	Passive in practice	Tutor directed	Supervisor directed	Learner directed	Mentor and mentee directed with repeated exchanges

### Appendix 2

**Table 5:** Summary of the goals and processes of each educational role

	Role modelling	Teaching & tutoring	Supervision	Coaching	Mentoring
**Purpose**	**Demonstrate positive behaviours**, such as - Professionalism [55, 100, 108] - Communication, collaboration and teamwork, management [55] - Admitting to errors, lifelong learning, humanistic skills and the patient-physician relationship [55, 56, 63, 110])	**Acquisition of standardized knowledge and skills**, training for clinical competency [65–71], guided by formal teaching structure [66, 69]	**Ensure trainees attain a minimum standard for safe practice** with a focus on patient safety [93, 96, 98, 99, 104, 122, 123], good patient care [92, 93] and clinical conduct [93, 95, 99, 124]: - Provision of effective training [92, 93, 96, 99, 104, 123] and monitoring, - Personalized supervisor feedback [94, 99] - Gradual independence of trainees [124] towards their professional growth and development [92, 93, 122]	**Maximize the trainee’s potential in a highly specific skill** [82, 84], involving complex objectives such as: - Communication skills [75, 77–79, 87, 125] - Psychological well-being skills [72, 73, 80, 81] - Clinical skills [74, 82, 83, 85, 86, 88–91, 126] - Use of evidence-based medicine [84] - Self-regulated learning skills [127] - Coaching pedagogy development skills [111]	**Professional and personal development**
**Process**	**Role modelling by trainer** (conscious/explicit or unconscious/accidental), **pertinent to the role of a physician** [55, 100, 107, 108] 1. **Observation** – Trainees observe the qualities and behaviour of role models [55, 100, 107, 108] 2. **Reflection and judgement** – Following observation, they make a judgement regarding whether the perceived behaviours are positive or negative [54, 59, 63, 100, 107, 108, 110, 128]. 3. **Emulation** – The trainee then ‘imitates’ or ‘mimics’ actions deemed beneficial and suitable to his or he own role as a physician [53, 100, 109, 110, 112]. 4. **Experimentation** – The trainee adopts an ‘iterative process’ to hone positive behaviour [64, 100, 110]. 4. **Assimilation by trainee** – Trainees incorporate behaviour they have been exposed to shape their own unique identity [53, 55, 64, 100].	1. **Initiation** – time scheduling for lessons [67] and student selection [67, 68] 2. **Preparation** –Teaching resources [65, 105] and tutor/teacher training [65, 68] 3. **Teaching** – Experiential learning [65, 67, 69, 70, 106] and facilitated discussion and presentations [65, 70, 71, 106] 4. **Feedback** – Subjective tutor/teachers’ feedback for students [66, 67, 105], Objective student assessment [66, 70, 71] and Program Evaluation by student [65, 68, 69]	1. **Initiation** – usually assigned and mandatory In most cases, supervision is initiated with the supervisor being assigned to oversee a particular junior doctor [96, 123, 129]. In some cases, supervision can be initiated by trainees and residents [129, 130]. 2. **Goal setting** – Supervisors bear more responsibility in identifying needs of learners, with less emphasis on reflection by trainee compared to coaching as beginners need direction. 3. **Observation/ Evaluation** The duration and nature of supervision varies across the various accounts given the lack formal frameworks in most programs [92, 98, 104, 123]. 4. **Feedback** The quality and efficacy of a supervisory relationship is assessed upon resident’s feedback [92], supervisor’s feedback [92, 95] and organizational evaluations [104].	**Coaching tends to be voluntary, highly structured** with targeted skills assessment and specific and individualized feedback [74–77, 79, 81–91, 111]. 1. **Initiation** 2. **Shared goals** – either pre- determined by curriculum or by trainee 3. **Observation** 4. **Individualized feedback and demonstration** Multisource feedback to complement the feedback from coaches and provide a wider insight of the trainees’ strengths and weaknesses, help develop specific goals and enhance strategies for improvement [79, 125]. 5. **Reflections** Four levels of reflections include cognitions (what came up into your mind?), emotions (what did you feel?), physical reaction (how did you feel?) and behaviour (observed verbal and non-verbal reaction) [75]. 6. **Practice** 7. **Repetition** To sustain the skills acquired, a critical part of coaching focuses upon ensuring that trainees monitor their practice, learn how to continue to improve and take responsibility for sustaining the gains made [85, 88].. 8. **Improvement** 9. **Mastery**	1. **Initiation** (assigned/matched) 2. **Goal setting** 3. **Developmental process** 4. **Realignment** 5. **Friendship**
**Nature of process**	- Formal and Informal - Passive and active - Conscious and unconscious - Less intentional	- Professional goal specific - Student-centric - Formal, standardized - Teacher-student dependent - Supportive - Evaluative	- Formal - Supportive - Evaluative	- Formal - Supportive - Evaluative	- Formal - Intentional
**Problems faced**	While good teaching skills correlates well with positive role modelling, it does not always indicate efficacious role model status, which emphasizes on specific nature of role modelling [101, 107, 131]. 1. **Lack of intentional role modelling by trainers** [53, 56, 58, 59, 62, 100, 107, 108], with good self-awareness 2. **Ineffective training on role modelling** to address incompatible personal traits of trainers and trainees [52, 58, 63] 3. **Poor self-awareness of trainees** [59, 64, 100, 107, 110], prone to emulating negative behaviours without careful judgement and reflection [59, 64, 100, 107, 110]	1. **Poor program structure** with inconsistent teaching guidelines [65], teaching qualities [69] and a lack of formal structure of tutoring [106] 2. **Individualized learner’s needs are unmet** due to inability to accommodate to variable student personality, knowledge and skills [65, 66, 68], and dissonance in teacher-student learning needs [70] 3. **Inaccurate program evaluation** Self-rated outcomes and teaching rates are not predictive of students’ performance [67, 68]. There is a paucity of objective measure of learning behavioural competencies [68, 70].	1. **Inaccurate program evaluation and poor program structure** [93, 122] due to diverse perceptions of supervision practices [92, 98, 99, 104, 123, 130], inaccurate assessment of trainee’s needs and skills [93, 104, 122], lack of consistent, validated and objective outcome measures [92, 99, 104] 2. **Lack of supervisor training** with difficulty relating to the learners, and meeting their specific needs [92, 122]. 3. **Supervisor burnout** with lack of protected time, interest, and presence of competing commitments [122]. 4. **Suboptimal learning environment** [92, 99, 123, 129, 130] with lack of supervisory feedback [132], fear of supervisory judgement, or loss of autonomy over learning [95]	1. **Unsupportive coaching environment** with conflicting educational roles as a coach, teacher, guide, and evaluator, deterring trainees from being genuine with their concerns [90].	
**Role of Host Organization**	1. **Developing role modelling-specific faculty development programs** [53, 55, 56, 62, 63, 107, 110, 115, 131] 2. **Gathering feedback from trainees** (such as through RoMAT questionnaire) to improve trainer’s status as role model [54, 128]		1. **Developing structured and specific training program** with established training process [96, 123, 129] to ensure oversight of clinical training [95].	1. **Creating individualized safe environment** to facilitate honest sharing [90] of weakness by matching with coaches of complementary characteristics with trainees [91] without any involvement in evaluating trainees [73].	

## Abbreviations

BEME: Best Evidence Medical Education
CoP: Codes of Practice
STORIES: Structured approach to the Reporting In healthcare education of Evidence Synthesis
